# Dye labelled monoclonal antibody assay for detection of Toxic Shock Syndrome Toxin -1 from *Staphylococcus aureus*

**Published:** 2011-12

**Authors:** Khojasteh V Javid, HA Foster

**Affiliations:** 1 Department of Bacteriology, Guilan University of Medical Sciences, Faculty of Shahid Beheshty, Rasht, Iran; 2 Center of Parasitology and Disease Reserch, School of Environment and Life Sciences, University of Salford, The Crescent, Salford M5 4WT, UK

**Keywords:** Monoclonal antibody, *Staphylococcus aureus*, TSST-1, Colloidal dye, ELISA

## Abstract

**Objective:**

The aim of study was to develop a rapid assay, dye labelled monoclonal antibody assay (DLMAA), using non-radioactive organic synthetic dyes for identification of Toxic Shock Syndrome Toxin-1 (TSST-1) producing strains of *Staphylococcus aureus*.

**Materials and Methods:**

The assay protocol required only two simple steps; addition of TSST-1 antigen to a nitrocellulose membrane and then adding a colloidal dye labelled antibody (D/A) suspension detection reagent.

**Results:**

The sensitivity and specificity of the assay was determined relative to positive and negative strains compared to an ELISA assay. Overall 100% agreement was found between both assays. The sensitivity for detection of TSST-1 was 30 ng.

**Conclusion:**

The DLMAA did not require handling and disposal of radioactive materials. It is a rapid qualitative technique for detection of TSST-1 toxin at room temperature within a short time.

## INTRODUCTION

Toxic Shock Syndrome Toxin -1 (TSST-1) is an extracellular superantigen toxin produced by some strains of *Staphylococcus aureus*. TSST-1 is one of the causes of Toxic Shock Syndrome (TSS). Toxic Shock Syndrome is a rare but serious consequence of burn wound infection and other staphylococcal infections and also is a disease that affects multiple organs and may be fatal (1). It is characterised by rapid fever, arterial hypotension, diffuse cutaneous rash, circulatory failure, vomiting, diarrhoea, myalgia, epidermis scaling, hypo-albuminemia and organ failure (multiple organ dysfunction syndrome– MODS) (2). If the symptoms or infection are not properly treated soon after the onset of symptoms, a fatal shock may develop within 24 h (3). Neill *et al*. found that approximately 20% of all *S. aureus* isolates produced TSST-1 (4). Approximately, 55% of strains of *Staphylococcus aureus* isolated from a paediatric burns unit produced at least one superantigen toxin including TSST-1and enterotoxins A-D (5). At the present, in order to distinguish between TSST-1 producing and non-TSST-1 producing strains of *S. aureus*, methods such as ELISA (6) and immune-blot assay are available (7). The gene for TSST-1 (*tst*) can be detected using PCR but not all strains containing the *tst* gene express TSST-1 (8). It has been reported that almost all strains (90%) from patients with menstrual TSS synthesise TSST-1, whereas only 50-60% of isolates from non-menstrual cases of TSS produce this protein (9). PCR is also an expensive technique. Some of these methods are relatively time consuming. They require at least 24 h for interpretable results and some also require special reagents or equipment. The ELISA method is more sensitive and specific than the other methods, but it is labour intensive and is more expensive (10). The development of a rapid assay for TSST-1 production would speed up identification of toxin producing strains. Gribnau *et al*. suggested that organic-synthetic, hydrophobic colloidal dye particles, and non-radioactive dye labels could be easily prepared from a wide range of commercially available disperses dyes (11). These could be used to label antibodies by simple physical adsorption and several detection methods can be applied to the final determination of the label such as visual observation and fluorimetry. The colloidal dye labelling of monoclonal antibodies is an alternative to current types of labelled immunoassays (12). Recent developments using various labels now enable the detection of specific TSST-1 antigen using the corresponding species-specific monoclonal antibodies (13).

In the present study, readily available disperse dyes were used for dying monoclonal anti-TSST-1 antibodies and applied for TSST-1 antigen detection using fine aqueous suspensions of dye-labelled antibodies at room temperature within a short time (4 hours).

## MATERIALS AND METHODS

**Organisms and culture conditions.** The *S. aureus* strains used in this investigation were: T1 (TSST-1 positive FR 1187S Courtesy of D. Taylor, University of Leeds, originally from Bergdoll), T2 (TSST-1 producer, West Lancashire Health Authority), T3 (TSST-1 producer, West Lancashire Health Authority), T4 (TSST-1 producer, confirmed case of TSS, Bury, UK) and T5 (TSST-1 producer, Bury, Health Authority), B68 (Enterotoxin A producer), B19 (Enterotoxin B producer), B27 (Enterotoxin C producer) and B78 (non-toxin producer). All strains were supplied by Professor V. Edwards-Jones, Manchester Metropolitan University, Manchester, UK.

One loop of *S. aureus* was streaked to give single colonies on Brain Heart Infusion (BHI) agar (LabM) and incubated overnight at 37°C. A few single colonies from the culture plate were suspended in saline to give turbidity equal to that of a McFarland 0.5 standard. One ml of this suspension was inoculated into 100 ml BHI broth in a 250 ml conical flask and incubated overnight at 37°C in a water bath shaker at 150 rpm. Ten ml of the culture was centrifuged at 25, 000×g at 4°C for 20 min. The supernatant fluid was removed and stored at −70°C for toxin assay.

**Assay of enterotoxins (A-D) and TSST-1.** The supernatant fluids were tested for the presence of enterotoxins and TSST-1 using Reverse Passive Latex Agglutination (RPLA) test kits from Oxoid Unipath (TST-RPLA, TD 940A for TSST-1 and SET-RPLA, TD900A for enterotoxins) according to the manufacturers specifications. Briefly, phosphate buffered saline (PBS, 25 µl) containing 5% bovine serum albumin was dispensed in each well of two rows in a 96 well conical bottom microtitre plate. Supernatant fluid (25 µl) was added to the first and second well of each row and doubling dilutions were performed to give titres ranging from 1:2 to 1:256. 25 µl of latex suspension sensitised with specific antibodies against TSST-1 or enterotoxins was added to each well in the first row. Twenty-five microliters of control latex suspension sensitised with non-immune rabbit immunoglobulin was added to each well of the second row. After thorough mixing, the plates were incubated in a humidified box for 18-20 h at room temperature. The titres were expressed as the reciprocal of the last dilution which gave an agglutination reaction (lattice). The control row showed a negative reaction (pellet) to indicate there was no non-specific reaction.

**Specificity of monoclonal antibody.** The superna-tant fluid of TSST-1 toxins (strains T1-T5 from Table 1) was tested at dilutions from 1:20 to 1:640 and the optimal working dilution was determined to be 1:100. Flat-bottomed microtitre plates (Immulon II, Dynatech) were coated with a 100 µl of this dilution or 100 µl of 10 µg ml^−1^ lyophilised TSST-1 toxin obtained from the TST-RPLA kit in 0.1 M carbonate-bicarbonate buffer (Na_2_CO_3_ and NaHCO_3_ pH 9.6) by incubation overnight at 4°C. Unbound toxin was removed by washing three times with 0.15 M phosphate buffered saline pH 7.2 containing 10 ml l^−1^ Tween 20, (PBS-T). The wells were blocked with 5% (w/v) powdered milk in PBS-T (150 µl/well) for 1 h at room temperature. The plates were washed 3x with PBS-T and incubated with100 µl of a 1 µg ml^−1^ solution of the specific anti-TSST-1 monoclonal antibody (Mab; 23.3, 1 mg/ml, was kindly donated by Dr H. Tranter, Centre for Applied Microbiology and Research, Porton, Salisbury, Wiltshire, UK, and 1:1000 was used as the working dilution) for 1 h at RT. The plates were washed 3x with PBS-T and 100 µl of a 1:2000 dilution of peroxidase conjugate added (Sigma, A6029) in PBS-T and incubated for 1 h at RT. The plates were again washed 3x with PBS-T, then 100 µl of 100 µg ml^−1^ 3,3’,5,5’-tetramethylbenzidine free base (Sigma, T5525) in phosphate-citrate buffer pH 5.0, containing 0.012% v/v hydrogen peroxide was added to each well and the plates were incubated at RT for 30 min. The absorbance of the solution in each well was read at 630 nm using a Dynatech ELISA plate reader. The same procedure was performed using alkaline phosphatase conjugate (Sigma, A6029) instead of peroxidase conjugate.

**Table 1:** Specificity of detection of TSST-1 from *Staphylococcus aureus* strains using ELISA and DLMAA.

Strain and toxin produced	Detection of TSST-1	
	ELISA	DLMAA
T1 (TSST-1)	+	+
T2 (TSST-1)	+	+
T3 (TSST-1)	+	+
T4 (TSST-1)	+	+
T5 (TSST-1)	+	+
B 68 (SEA)	−	−
B19 (SEB)	−	−
B27 (SEC)	−	−
B78 (Non-toxin producer)	−	−
Purified TSST-1	+	+

For some experiments the possible interfering effects of protein A were prevented by adsorption onto normal serum. Culture supernatant of *S. aureus* toxin producing strains, TSST-1, enterotoxin A, B, C and non-toxin producing strains (negative control) were prepared in BHI broth and were pre-treated with 10% normal rabbit serum to remove protein A.

**Dye-labelling: Preparation of dispersed dye.**Six samples of textile disperse dyes (red BF 200, luminous red G, yellow 3GE 200, luminous yellow GN, brilliant blue BG-CF and dark blue 3RT-CF) were kindly donated by BASF PLC, Cheshire SK8 6QG, United Kingdom. Dye particle suspensions were prepared using a washing and centrifugation procedure previously described (11). Briefly, 1 g of each dye was suspended in 20 ml distilled water and washed 4x by centrifugation at 20,000×g for 30 min and resuspension until the supernatant was colourless. A final centrifugation was performed at 125×g for 30 min in order to remove large particles. The suspension was carefully decanted and 0.01% (v/v) thiomersol was added as a preservative. The maximum adsorption wavelength (Eλ_max_) was determined using a Perkin-Elmer Lambda 5 UV/VIS scanning spectrophotometer using dye particle suspensions solubilised in ethanol. Dye particle suspensions were diluted and standardised in distilled water to give λ_max_=1. The Mab to TSST-1 toxin was linked to the colloidal particle dyes (dye/Mab) using a modification of a previously described technique (11, 14, 15). The optimum conditions for dye and Mab were determined by using combinations of buffer system (10 mM sodium phosphate buffer containing 2.7 mM NaCl, 33.3 mM sodium phosphate buffer containing 0.125 M NaCl) at two different pH (4.5 and 7.5), four dye concentrations (A=10, 20, 30 and 50) and three antibody concentrations (50, 80 and 100 µg/ml).

**Preparation of dye antibody complex.** Appropriate volumes of dye/Mab were incubated in 10 mM sodium phosphate buffer (Na_2_HPO_4_.12H_2_O and NaH_2_PO_4_.2H_2_O) containing 2.7 mM NaCl, pH 7.5 for 2 hours to allow for absorption to colloidal dye particles at 4°C. The solution was then spiked with 1/5 volume of a 20% bovine serum albumin (BSA) solution in 5 mM NaCl pH 7.5 and incubated overnight at 4°C to stabilise the dye particle surfaces. The dye/Ab reagent was centrifuged at 17,000×g and the pellet was resuspended in 33.3 mM Na_2_HPO_4_.12H_2_O and NaH_2_PO_4_.2H_2_O buffer pH 7.5, containing 0.125 mM NaCl, 5% BSA and 0.01% thiomersol.

**Membrane strips and blocking reagents.** Amersham Hybond-C and Amersham Hybond C-Extra (Amersham International), which contains an inert matrix that makes the membrane more robust, and blocking reagents (30 g l^−1^BSA or 5% dried skimed powdered milk in PBS) were used to block unbound protein. Subsequent tests used phosphate buffered saline plus milk (PBSM) for blocking with 45 min incubation for Amersham Hybond-C (Nitrocellulose, Cat. No. RPN 2109).

**Dye labelled monoclonal antibody (DLMA) test method.** To standardise the DLMA assay, 3 µl of different concentrations (50 µg ml^−1^, 20 µg ml^−1^, 10 µg ml^−1^; equivalent to 150, 60, 30 ng toxin per strip respectively) of purified TSST-1 toxin (Sigma,T5662) as a positive control and 3 µl of supernatant fluid of strains T1-T5 were added as spots of at one end of each membrane strip and left to dry for at least 1 h. The strips were placed in Petri dishes. The strips were washed under tap water for 15 sec and the remaining protein binding sites blocked by the addition of PBSM pH 7.4 and incubation for 45 min. The strips were washed under tap water for 15 sec and incubated with dye/Mab reagent for 2 h at RT. After this time the strips were washed once again under tap water for 15 sec, dried and photographed.

## RESULTS

**Fig. 1:**
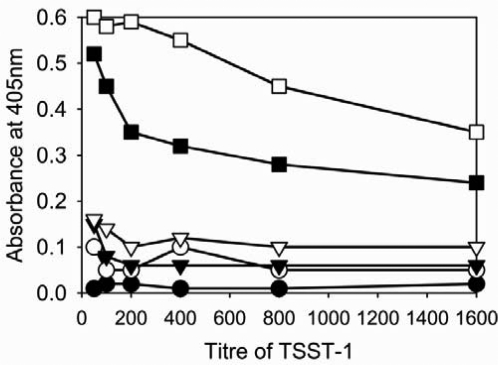
Sensitivity and specificity of the monoclonal antibody to TSST-1 in culture supernatant fluids from *Staphylococcus aureus* using ELISA and alkaline phosphatase conjugate. Supernatants from *S. aureus* strains: non-toxin producer •, SEA ○, SEB ▾, SEC s ▿,TSST-1 ▪ and purified TSST-1 □

**Fig. 2:**
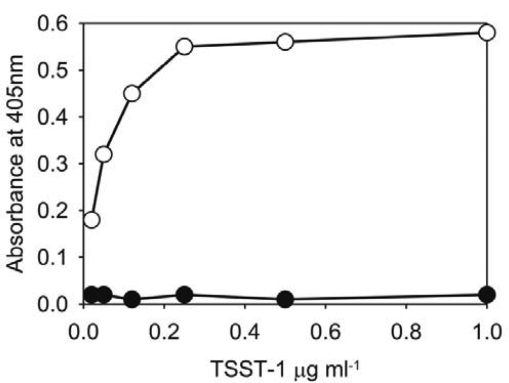
The detection of different concentrations of purified TSST-1 with monoclonal antibody using ELISA Supernatant from non-TSST-1 producer •, purified TSST-1 ○.

**Specificity and sensitivity of ELISA.** The specificity of the monoclonal antibody was determined by testing supernant fluids from TSST-1 positive, TSST-1 negative strains of *S. aureus* and purified TSST-1 (Sigma, T5662) at concentration of 50 µg/ml, 20 µg/ml, 10 µg/ml, 5 µg/ml and 2.5 µg/ml and the results are shown in Fig. 1). There was a positive reaction to the purified TSST-1 and the culture supernatant fluid from the TSST-1 producing strain but not to those from the enterotoxin A, B or C producers or the non-toxin producing strain, confirming the specificity of the Mab. The sensitivity of the ELISA method for identification of TSST-1 was determined by using different concentrations of purified TSST-1 toxin and was 3 µl from a 10 µg/ml solution (30 ng/ml) of toxin (Fig. 2).

**Detection of the dyes.** The optimum wavelength for quantitation of each dye was determined by measuring the wavelength for maximum absorption (Eλ_max_) of appropriate dilutions using a scanning spectrophotometer (Pye-Unicam SP6 UV/Visible). Eλ_max_ for each dye was: yellow 3GE, 441.6; luminous yellow, 460.4: luminous red, 548.6; dark blue 3RT, 587.2; brilliant blue, 667.8 and red BF, 517.4. Dye suspensions were then standardized at Eλ_max_.= 30.

**Fig. 3:**
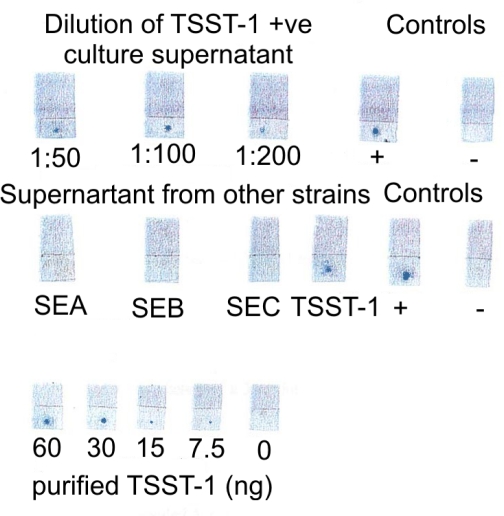
Sensitivity and specificity of optimised dye-labelled monoclonal antibody to detect TSST-1 Top row, detection of TSST-1 in different dilutions of culture supernatant from a TSST-1 positive *S.aureus* strain; middle row supernatant fluid of enterotoxin A-C producing strains compared to a TSST-1 toxin producing strain; bottom row detection of different quantities of purified TSST-1.

**Optimisation of dye labelled assay.** Experiments were carried out to determine the optimum concentration of disperse dyes absorbed to the Mab. Fifty, 80, 100 and 200 µg of the Mab were added to 1 ml of different dye suspensions with absorbance (Eλ_max_=10, 30 and 50). Nitrocellulose strips with spots containing different amounts of purified toxin were then incubated with the dye-antibody mixtures washed and dried. Adequate colour development was observed using 100 µg Mab in 1 ml of dye with E λ_max_=30 and the results are shown in Fig. 3. Clear spots were visible with 3 µl of supernatant fluid of the TSST-1 toxin producing strains or 3 µl of 10 µg ml^−1^ of the purified TSST-1 toxin (30 ng) as capturing antigens. This reagent was used in subsequent experiments.

Preliminary experiments showed that clearer spots were produced with brilliant blue BCG than with the other dyes. Red BF, Luminous Yellow and Yellow dyes adequately labelled the Mab, but the dots were sometimes difficult to visualise. Positive dots were obtained using Dark Blue but there was some background staining around the dots on the membrane. Brilliant blue BCG was used for subsequent experiments.

Different concentrations of purified TSST-1 toxin (20, 10, 5 and 2.5 µg ml^−1^) were applied to nitrocellulose strips and the test repeated using the optimised reagent. The results showed that clear stained spots were visible at concentration of 10 µg/ml of commercially purified TSST-1 toxin which was selected as positive control for further tests.

**Fig. 4:**
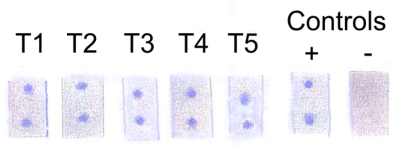
Assay of culture supernatant fluids from five different TSST-1 (T1, T2, T3, T4, T5) producing strains using the optimised dye labelled monoclonal antibody.

**Specificity of dye labelled assay.** The specificity of DLMA assay was evaluated using membrane strips spotted with 3 µl of supernatant fluid from enterotoxin A, B and C producing strains, a non-toxin producing strain of *S. aureus* and purified TSST-1 toxin (Fig. 4). Supernatant fluids from the enterotoxin producers and the non-toxin producer gave negative results. Positive spots were obtained with the purified toxin and culture supernatant fluids from five different TSST-1 producing strains of *S. aureus* (Fig. 4).

**Comparison of results using ELISA and dye labelled assay.** Nine samples of culture supernatants from non-toxin producing and toxin producing strains were tested using ELISA and DLMA assays (Table 1). One hundred percent agreement was found between the two assays.

## DISCUSSION

The results show that dye-labelled antibodies can be used to detect TSST-1. The non-radioactive, organic-synthetic dye label can be easily prepared from a wide range of commercially available disperse dyes. The assay procedure developed here is economical, with a minimum number of reagents and actions per assay. As compared to ELISA, for example, dye labelled antibodies have the advantage that the enzyme (substrate-chromogen) incubation can be omitted and the results can be read visually without any special equipment. Furthermore, it is rapid and suited for use in unsophisticated laboratories and can be used for epidemiological investigations in the field.

Parsonnet *et al*. described a competitive ELISA capable of quantitating 30 ng of TSST-1 per ml in *S. aureus* culture supernatants (16). This assay was not influenced by protein A but did cross-react minimally with staphylococcal enterotoxins A, D, and E. The DLMA assay used here showed no cross reaction with enterotoxins. Furthermore, a competitive ELISA with enzyme-labelled antigen required purified TSST-1 in relatively large amounts for preparation of the enzyme-antigen conjugate. A more important disadvantage of this method is that the competitive ELISA might not be readily adapted. This is due to the need to incubate enzyme-labelled TSST-1 directly with biologic fluids or tissue extracts which themselves may contain proteases and may significantly alter the activity of the enzyme conjugated to TSST-1. The test described here does not require handling and disposal of radioactive materials. It is a rapid qualitative technique for detection of TSST-1 at room temperature within a short time (4 h). The results showed that clearer spots were produced with brilliant blue BCG than with the other dyes but all dyes tested gave visible spots.

These observations support previous work that demonstrated disperse dyes as suitable for DIA reagents need to be screened and that selection of a particular dye depends on the antibody preparation (14). Textile disperse dyes are produced widely in the chemical industry and are typically marketed in kilogram quantities. Consequently costs for the production of DLMAA reagents are negligible compared with products for Dot-blot or ELISA. The sensitivity of DLMA and ELISA were comparable and both assays and could detect TSST-1 at concentration of 30 ng per 3 µl. ELISA is an accepted method for detecting TSST-1 producing *S. aureus* (6, 10) but cross reaction with Protein A can interfere with the ELISA by binding to the coating or enzyme conjugate (17). The influence by protein A can be eliminated by pre-treatment of samples with 10% rabbit serum. No components in the BHI medium used here showed interference with the assay. Research by Kvo and Silverman on detection of staphylococcal enterotoxin in food showed that sensitivity can be obtained using either phosphatase or peroxidase labelled antigen for SEA or SEB but reported the possibility that endogenous peroxidase could be present in the culture media (18). In the present study the use of alkaline phosphatase conjugate eliminated the possibility that endogenous peroxidase could be present in the culture media. Furthermore, the use of alkaline phosphatase conjugate instead of peroxidase conjugate gave more consistent results in the assay.

The DMLAA method described here could be applied for detection of TSST-1 produced from strains isolated from patients with TSS either in clinical practice or in fields where simple technology is required. The method presented here could potentially be applied for qualitative toxin detection tests for a variety of *S. aureus* enterotoxins using monoclonal antibodies to each toxin labelled with different coloured dyes.
